# Acute myocardial infarction after inactivated COVID-19 vaccination: a case report and literature review

**DOI:** 10.3389/fcvm.2023.1123385

**Published:** 2023-05-30

**Authors:** Weimei Ou, Bin Wang, Guoming Zhang, Licheng Ding, Zhixian Liu, Kaimin Wu, Guangfeng Sun, Chengmin Huang, Zhaokai Li, Shuyuan Feng, Rui Gao

**Affiliations:** ^1^ Department of Emergency, Xiamen Cardiovascular Hospital of Xiamen University, School of Medicine, Xiamen University, Xiamen, China; ^2^ Institute of Cardiovascular Diseases, Xiamen Cardiovascular Hospital of Xiamen University, School of Medicine, Xiamen University, Xiamen, China

**Keywords:** acute myocardial infarction (AMI), COVID-19 vaccination, kounis syndrome (KS), coronary heart disease (CHD), coronary thrombosis

## Abstract

A number of vaccines have been developed and deployed globally to restrain the spreading of the coronavirus disease 2019 (COVID-19). The adverse effect following vaccination is an important consideration. Acute myocardial infarction (AMI) is a kind of rare adverse event after COVID-19 vaccination. Herein, we present a case of an 83-year-old male who suffered cold sweat ten minutes after the first inactivated COVID-19 vaccination and AMI one day later. The emergency coronary angiography showed coronary thrombosis and underlying stenosis in his coronary artery. Type II Kounis syndrome might be a potential mechanism, which is manifested as coronary thrombosis secondary to allergic reactions in patients with underlying asymptomatic coronary heart disease. We also summarize the reported AMI cases post COVID-19 vaccination, as well as overview and discuss the proposed mechanisms of AMI after COVID-19 vaccination, thus providing insights for clinicians to be aware of the possibility of AMI following COVID-19 vaccination and potential underlying mechanisms.

## 1. Introduction

The coronavirus disease 2019 (COVID-19) has become a pandemic since March of 2020. At the time of writing, more than 640 million cases have been confirmed, including more than 6.6 million cases of death (1). A number of vaccines have been developed and deployed and shown to be the most effective strategy to restrain the spread of COVID-19 (2). Up to date, more than 13 billion doses of COVID-19 vaccines have been administered globally (1). However, many kinds of adverse events (AE) brought to people's attention after COVID-19 vaccination, even though the safety and efficacy have been tested (3–11), for example, acute myocardial infarction (AMI), myocarditis, pericarditis and so on (12–14). Importantly, it is reported that people showed individual differences in humoral immune response depending on many factors such as age, sex, serostatus, and underlying comorbidities (15, 16). Therefore, the AE cases could differ from each other as well. In general, most adverse reactions were mild, with the most common symptoms being injection-site pain, fatigue, headache, myalgia, and nausea (6, 9, 17–19). Even though the incidence of serious AE was low (3, 4, 18, 20, 21), genuine concerns and attentions should be raised on these rare serious AE.

Traditional inactivated whole-virus COVID-19 vaccine is safe and efficacious to prevent COVID-19 pandemic (9, 18). Generally, AE after vaccination are neither frequent nor serious (9). However, rare AE such as acute myocardial infarction (AMI) are potentially life-threatening (22, 23). Herein, we present a case of an 83-year-old male who suffered allergic reactions developing to AMI ten minutes after the first inactivated COVID-19 vaccination in China. Coronary angiography (CAG) showed acute coronary thrombosis and underlying stenosis in the coronary artery. The potential mechanism of AMI after inactivated COVID-19 vaccination might be type II Kounis syndrome (KS), which was manifested as coronary thrombosis secondary to allergic reactions in patients with underlying asymptomatic coronary heart disease (CHD).

## 2. Case description

An 83-year-old man with a medical history of subtotal gastrectomy and cholecystectomy complained of cold sweat, dizziness, fatigue and transient loss of consciousness ten minutes after the first dose of inactivated COVID-19 vaccine (CoronaVac, 202106061Z, Sinovac Life Sciences, Beijing, China). He felt worse accompanied by chest tightness, diarrhea and a lowest blood pressure of 83/50 mmHg the following day, and was admitted to the emergency department (ED). The patient denied any history of CHD, hypertension, diabetes, renal dysfunction, asthma or allergic reaction. He also denied any cardiac or noncardiac symptoms before vaccination. The detailed medical history of the patient was showed in Supplementary Table S1.

Upon ED arrival, the physical examination showed the blood pressure was not very high with 98/66 mmHg, other vital signs were normal. Laboratory tests showed the markers of myocardial injury were elevated, with high sensitivity troponin-T level more than 2,000 (reference: 0-100) ng/L, myohemoglobin 291 (reference: 28–72) ng/ml, creatine kinase (CK) 2771.7 (reference: 50–310) U/L, CK-MB fraction 348.2 (reference: 0–19) U/L, D-dimer 1.09 (reference: 0–0.55) mg/L, and N-terminal pro-brain natriuretic peptide 1,770 (reference: 0–125) pg/ml. Infection indices of white blood cells [10.75 (reference: 3.5–9.5) × 10^9^/L], hypersensitive C-reactive protein [6.13 (reference: 0–3) mg/L] and procalcitonin [0.103 (reference: 0–0.05) ng/ml] were modestly increased. Other laboratory tests were within normal ranges.

**Figure 1:**
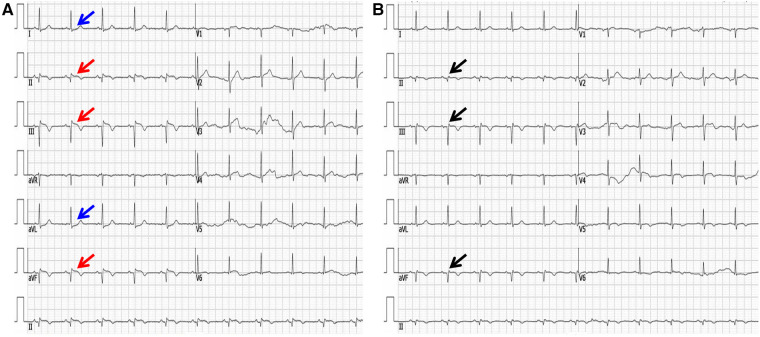
The electrocardiogram upon arrival (**A**) and one week later (**B**). **(A)** The red arrow shows ST segments were elevated in leads II, III and avF. The blue arrow shows ST segments were reciprocally depressed in leads I and avL. **(B)** The black arrow shows pathological Q waves in inferior leads, with ST segment recovery.

**Figure 2:**
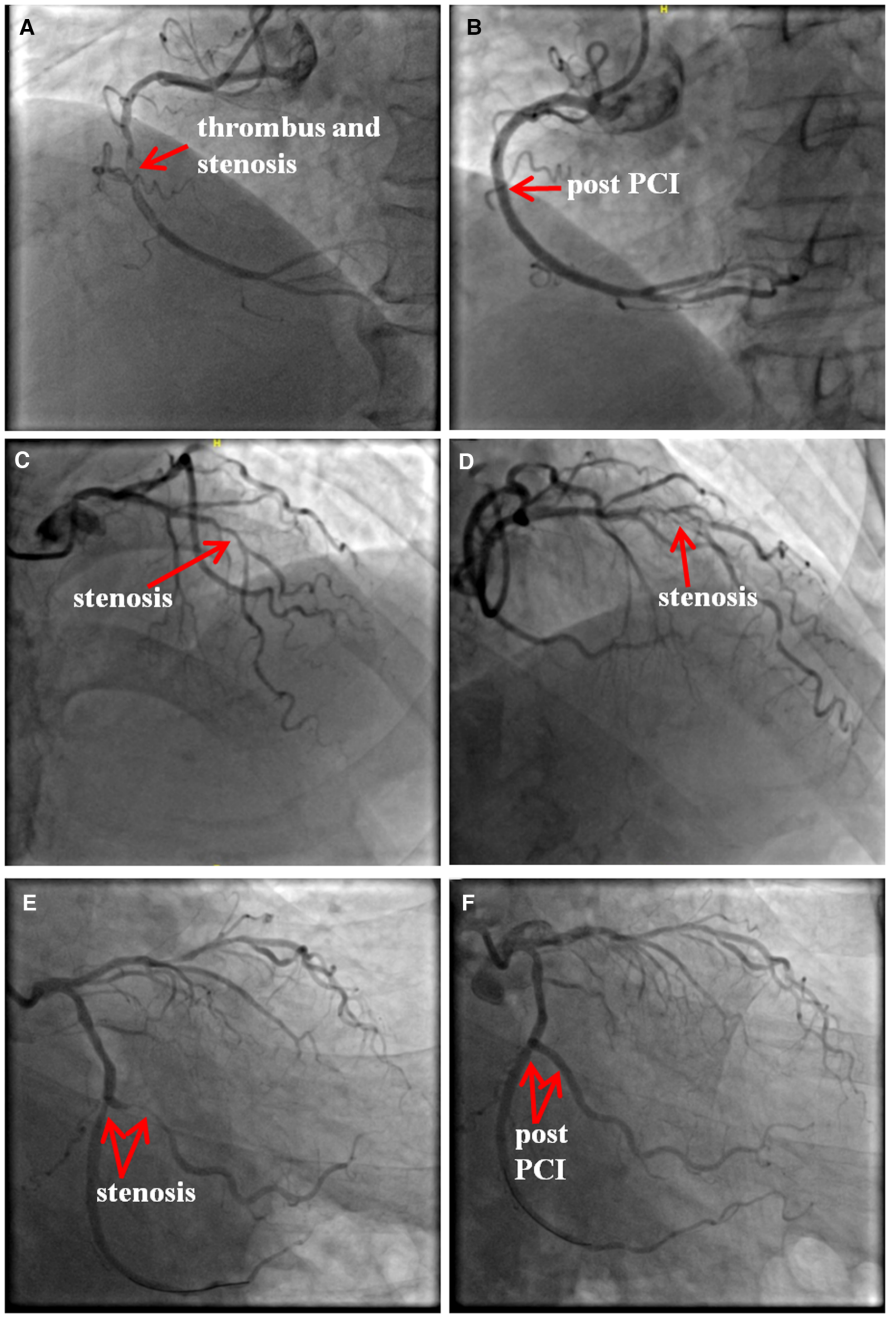
The results of coronary angiography. There was 95% stenosis in the right coronary artery (RCA) with a local thrombotic shadow (**A**), and two drug eluting stents (DES, Firebird 3.0 × 29 mm, Firebird 3.5 × 33 mm) were implanted into the RCA. (**B**) There was 80% stenosis in the left anterior descending coronary artery (**C,D**). There was 80%–90% stenosis in the obtuse marginal branch (OM) and 70%–80% stenosis in the distal LCX (d-LCX) (**E**), and a DES (Promus Element 2.25 × 20 mm) and a drug-coated balloon (Vesselin 3.0 × 16 mm) were implanted in the OM and d-LCX, respectively. (**F**) PCI, percutaneous coronary intervention.

On admission, the electrocardiogram (ECG) showed that ST segments were elevated in leads II, III and avF, with reciprocal depression in leads I and avL (Figure 1A). Transthoracic echocardiography revealed hypokinesia on the inferior and posterior walls, with a left ventricular ejection fraction of 54%. The CAG revealed 95% stenosis in the right coronary artery (RCA) with thrombotic shadow locally, 80% stenosis in the left anterior descending coronary artery, 70%–80% stenosis in the distal left circumflex coronary artery (d-LCX), and 80%–90% stenosis in the obtuse marginal branch (OM) (Figures 2A,2C–E).

**Figure 3:**
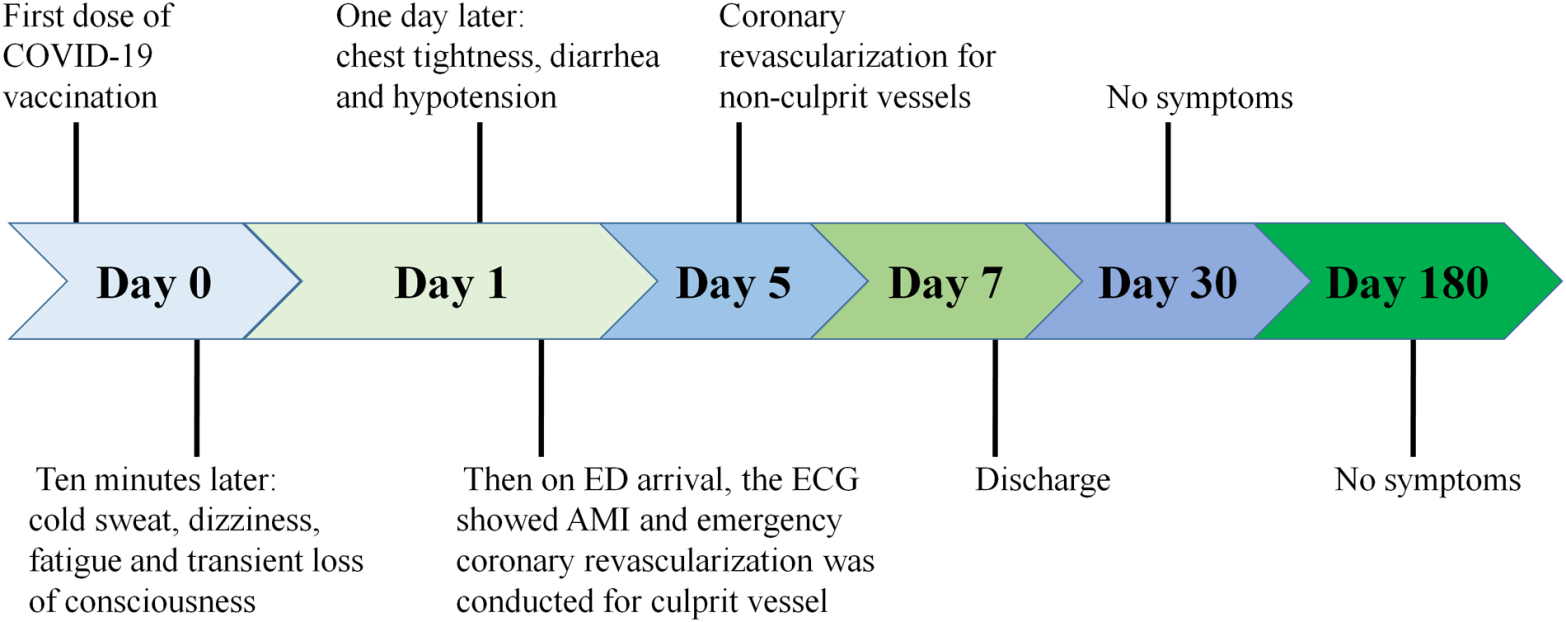
Schematic diagram showing the timeline from vaccination to the onset of AMI up until patient discharge.

Combined with the ECG result, RCA was considered as the culprit vessel. Therefore, thrombus aspiration was performed, and two drug-eluting stents (DES, Firebird 3.0 × 29 mm, Firebird 3.5 × 33 mm) were implanted into the RCA (Figure 2B). This was accompanied by oral drugs for secondary prevention (aspirin, ticagrelor, atorvastatin, metoprolol). Subsequently, the ECG showed pathological Q waves in the inferior leads with ST segment recovery (Figure 1B). Before discharge, a DES (Promus Element 2.25 × 20 mm) and a drug-coated balloon (Vesselin 3.0 × 16 mm) were implanted into the OM and d-LCX, respectively (Figure 2F). A week later, the abnormal cardiac injury markers were relieved. The patient was discharged from the hospital in good condition. Furthermore, at the 1-month and 6-month follow-up visits after discharge, he was doing well with no symptoms or abnormalities of cardiac markers. A schematic diagram showing the timeline from vaccination to the onset of AMI up until patient discharge was showed in Figure 3.

## 3. Literature review and discussion

In this part, we will summarize the reported AMI cases, as well as overview and discuss the proposed mechanisms of AMI after COVID-19 vaccination. From the searching of literature databases, 21 literatures were found regarding AMI following COVID-19 vaccination. The summary is shown in Supplementary Table S2. Generally, there are 30 AMI cases following COVID-19 vaccination including our case. The cases reported located all over the world including the US, European countries and Asian countries. The vaccines administered in these cases varied from mRNA vaccine (16 cases; Pfizer-BioNTech and Moderna), adenoviral vector vaccine (10 cases; AstraZeneca), to inactivated virus vaccine (4 cases; Sinovac), suggesting AMI is not the specific AE from one kind of vaccine. The mean age of patients is 64, indicating the incidence of AMI following COVID-19 vaccination is more likely among old population, which agrees with the results in a systematic study published in 2021 (12). Most of the cases showed symptoms within 24 h (76.7%; 23 out of 30 cases) and chest discomfort is the most common seen symptom in AMI cases (66.7%; 20 out of 30 cases) that should be paid more attention after COVID-19 vaccination, consistent with the finding in the systematic review published recently (24).

So far, there are some proposed underlying mechanisms for AMI following COVID-19 vaccination. The most probable explanation is KS, which is the concurrent occurrence of acute coronary syndromes with allergic reactions. To date, four variants of the disease have been documented: (1) type I: coronary spasm in patients with (nearly) normal coronary arteries; (2) type II: coronary thrombosis in patients with underlying asymptomatic CHD; (3) type III: stent-related allergic coronary events, with IIIa (stent thrombosis) and IIIb (in-stent restenosis); and (4) type IV: anaphylaxis-mediated AMI in patients with coronary grafts (25–28). In our case, the patient denied any medical history of CHD, but we found not only acute coronary thrombosis but also underlying stenosis in his coronary arteries, suggesting he should have pre-existing asymptomatic CHD that was not realized by himself. Given his symptoms occurred only ten minutes after the vaccination with no other triggers identified, accordingly, our patient was speculated with type II KS after inactivated COVID-19 vaccination, which was similar to a previous report of type I KS in Turkey (29). In addition, vaccine-induced thrombotic thrombocytopenia (VITT) associated thrombosis could be another possibility for AMI post COVID-19 vaccination, but only ten minutes is not enough to develop VITT, therefore this potential is very rare (22). Moreover, there was also a possibility that the vaccination and AMI were just coincident, though it was scarce. Given possible life-threatening results and lack recognition of KS, more attention should be drawn to KS by clinicians post COVID-19 vaccination.

**Figure 4:**
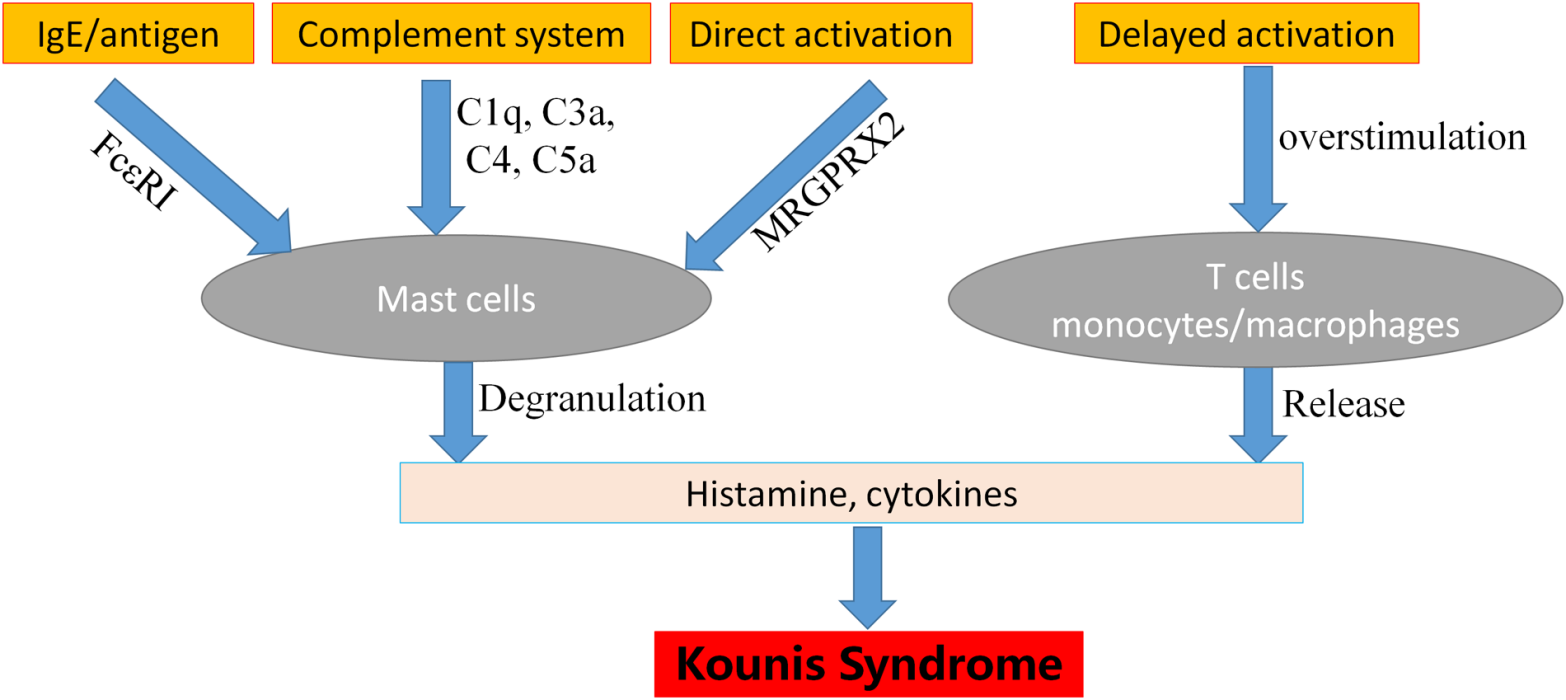
The mechanisms of Kounis syndrome pathophysiology. MRGPRX2: mas-related G protein-coupled receptor X2.

The pathophysiology of vaccine-induced allergic reactions could be derived from the following four kinds of mechanisms (30). (1) Reactions via the pathway of mast cell activation and degranulation as IgE/antigen through cross-linking of Fc*ε*RI on mast cells (31). This reaction typically occurs within minutes of exposure to the relevant allergen and always occurs within 4 h of exposure to the relevant allergen (32). This mechanism is confirmed by the specific IgEs detection and the increased levels of serum tryptase (30). (2) Non-IgE-mediated mast cell degranulation is another pathway that is performed via activation of the complement system, leading to the generation of anaphylatoxins C1q, C3a C4 and C5a. This complement pathway activation and positive biofeedback loops involving interleukin-5 (IL-5) and tryptase is also very common and should be considered (30, 33). (3) Life-threatening allergic reactions can be mediated via direct activation of the Mas-related G protein-coupled receptor X2 (MRGPRX2) that may activate mast cells via non-Fc*ε* receptors. In this pathway, the specific IgEs may remain undetected, and tryptase levels may be normal even in serious KS (30). This might explain the conditions in cases reported by Baronti et al. (34) that even the tryptase testing is negative, allergic reactions cannot be ruled out. (4) Hypersensitivity delayed reaction generally begins 48 h after vaccination and peaks between 72 and 96 h (35), which is cell-mediated and antibody independent, derived from overstimulation of T cells and monocytes/macrophages and releases of cytokines that cause inflammation, cell death, and tissue damage (30). Vaccines containing anti-microbial agents and ingredients, such as thimerosal and aluminum, can be followed by delayed reactions (32). A schematic diagram regarding pathophysiologic mechanisms of KS was shown in Figure 4.

Allergic reactions to vaccines are rarely attributed to the active vaccine itself, rather than excipients which constitute inert substances added to vaccines and other drugs to improve stability, increase solubility, improve absorption, influence palatability, or create a distinctive appearance (30). The viral vector Covishield vaccine contains aluminum hydroxide, and excipients similar to AstraZeneca vaccines such as polysorbate 80 (PS80) and disodium edetate dihydrate (ethylenediaminetetraacetic acid). The Moderna vaccine contains polyethylene glycol which is also shown in Pfizer-BioNTech vaccine, and tromethamine (also known as trometamol). The Sinovac (Coronavac) vaccine contains disodium hydrogen phosphate, sodium dihydrogen phosphate monohydrate, and sodium chloride (36). These excipients are also found in other vaccines such as influenza vaccine, and in creams, ointments, lotions, other cosmetics, various dental materials, as well as anticancer drugs which could sensitize their users (36). This situation has been reported by Fialho et al. that one case of AMI patient after COVID-19 vaccination from AstraZeneca who had a medical history of acute coronary syndrome after influenza vaccine was diagnosed with type III KS (37). In this case, both vaccines (COVID-19 and influenza) contain a common excipient PS80 (37). Even though the skin tests with intravenous amiodarone, that contains PS80, were negative, which rule out the IgE-mediated PS80 reaction, but cannot exclude PS80 non-IgE mediated hypersensitivity reactions (37, 38).

Even though multiple AMI cases have been reported occurring within minutes to hours after COVID-19 vaccination, there is still no enough evidence to show the direct causal relationship between AMI and COVID-19 vaccination. Since AMI is a commonly occurring disease in old people, whether the cases recorded are due to COVID-19 vaccination or just coincidence need further studies to elucidate.

Preliminary clinical trials in the Food and Drug Administration briefing documents indicated that the incidence rate of AMI was 0.03% and 0.02% after receiving Moderna or Pfizer vaccines, respectively (39). Compared with the total number of vaccine doses given, the incidence of AMI is really very rare. But it is potentially life-threatening. Clinicians need to be aware of this situation that might present after the COVID-19 vaccination.

## 4. Conclusion

In recent two years, vaccines have been deployed to restrain the spreading of COVID-19. AMI is a kind of rare AE following COVID-19 vaccination. In this article, we are reporting an AMI case that showed symptoms only ten minutes after COVID-19 vaccination with no other triggers identified. KS could be a potential mechanism in our case. Moreover, we summarize the reported AMI cases, as well as overview and discuss the proposed mechanisms of AMI following COVID-19 vaccination, thus providing insights for clinicians to be aware of the possibility of AMI following COVID-19 vaccination and potential underlying mechanisms.

## Data Availability

The original contributions presented in the study are included in the article/**Supplementary Material**, further inquiries can be directed to the corresponding author/s.
