# Supplementary material for: Acute myocardial infarction after inactivated COVID-19 vaccination: a case report and literature review
Source: Front Cardiovasc Med. 2023 May 30;10:1123385. doi: 10.3389/fcvm.2023.1123385 (PMC10262047; doi:10.3389/fcvm.2023.1123385)
Supplement: Supplementary file 1 [file Table1.docx]

Supplementary Material

**Supplementary table 1.** Main clinical characteristics and detailed medical history of the patient.

| Age | 83 years old |
| --- | --- |
| Sex | Male |
| Lifestyle |  |
| Smoking | No smoking for 40 years |
| Drinking | Drink a little occasionally |
| Stay up | No |
| Fractiousness | No |
| Past medical history |  |
| Heart disease | No |
| Diabetes | No |
| Hypertension | No |
| Hyperlipidemia | No |
| Obesity | No |
| Renal dysfunction | No |
| Hypohepatia | No |
| Asthma | No |
| Allergic history | No |
| Angioedema | No |
| Chronic granulomatous disease | No |
| Surgery | Subtotal gastrectomy more than 40 years and cholecystectomy more than 10 years |
| Cardiac symptoms before vaccination | No |
| Noncardiac symptoms before vaccination | No |
| Maintenance medications | No |
| Emotional triggers | No |

**Supplementary table 2.** Myocardial infarction cases following COVID-19 vaccination.

| Author | Country | Vaccine administered | Age/gender | Vaccination to symptom interval | Symptom | Medical history | Diagnosis | Outcome |
| --- | --- | --- | --- | --- | --- | --- | --- | --- |
| Barsha et al., 2021 | Bangladesh | Moderna COVID-19 vaccine; 1^st^ dose | 77/M | 12 hours | After 12 hours of vaccination, he developed fever, went unconscious after 36 hours. | DM2, peripheral neuropathy, and chronic obstructive pulmonary disease. | Encephalopathy and NSTEMI | alive |
| Boivin and Martin, 2021 | USA | Moderna COVID-19 vaccine; 1^st^ dose | 96/F | 1 hour | Chest discomfort. | Poorly controlled hypertension and a DNR/DNI code status. | MI | alive |
| Chatterjee et al., 2021 | India | Covishield vaccine; 1^st^ dose | 63/M | 2 days | Uneasiness, dizziness and excessive sweating. | None. | STEMI | alive |
| Chiang et al., 2021 | Taiwan, China | ChAdOx1 nCoV-19 (AstraZeneca); 1st dose | 75/F | 8 days | Chest tightness and epigastric pain accompanied by tarry stool for two days. | Chronic renal failure; DM2 and coronary artery disease. | NSTEMI | alive |
| Flower et al., 2021 | UK | ChAdOx1 nCov-19 vaccine; 1^st^ dose | 40/M | 2-8 days | Transient myalgia and febrile symptoms. | Smoking and gastric ulceration. | MI secondary to VITT | alive |
| Lee et al., 2021 | Singapore | Pfizer-BioNTech COVID-19 vaccine; 1^st^ dose | 73/F | 2 hours | Palpitations and shortness of breath. | Hypertension. | MI with non obstructive coronary arteries | N/A |
| Maadarani et al., 2021 | Kuwait | COVID vaccine (AZD1222-Oxford University and AstraZeneca); 1^st^ dose | 62/F | 1.5 hours | Central chest pain. | DM, hypertension and dyslipidaemia. | STEMI | alive |
| Özdemir et al., 2021 | Turkey | Inactivated CoronaVac (Sinovac Life Sciences, Beijing, China) ; 1^st^ dose | 41/F | 15 min | Flushing, palpitation, dyspnea, and chest pain. | None. | Type 1 KS | alive |
| Sung et al., 2021 | USA | COVID-19 vaccine (mRNA-1273); 1^st^ dose | 68/F | < 24 hours | Gradual onset of left shoulder pain within 1 day after vaccination; later progressed to left-sided chest pain. | Smoker; hypertension; hyperlipidemia; prior Coronary Artery Disease. | AMI | alive |
| Sung et al., 2021 | USA | COVID-19 vaccine (mRNA-1273); 1^st^ dose | 42/M | < 24 hours | 4 days of chest and shoulder pain. | Hyperlipidemia. | AMI | alive |
| Tajstra et al., 2021 | Poland | Pfizer–BioNTech vaccine; 1^st^ dose | 86/M | 30 min | Unconscious, with clinical and hemodynamic signs of cardiogenic shock and recurrent bradyar-rhythmias. | Prostate cancer; paroxysmal atrial fibrillation. | STEMI | dead |
| Allam et al., 2022 | Greece | BNT162b1 mRNA COVID-19 vaccine (Pfizer, New York); 1^st^ dose | 64/M | 15 min | Chills, chest pain, pallor, diaphoresis, and hypotension. | STEMI and drug-eluting stent was implanted in the proximal LAD. | Anaphylaxis-induced stent thrombosis, or type III KS | N/A |
| Baronti et al., 2022 | Italy | BNT162b2, 1^st^ dose | 69/M | 24–48 hours | Unwitnessed; complained of shivering, thoracic, and upper limbs pain some hours before. | Chronic obstructive pulmonary disease (COPD); DM2; smoking. | MI | dead |
| Baronti et al., 2022 | Italy | BNT162b2, 2^nd^ dose | 58/M | < 24 hours | Unwitnessed; complained of thoracic pain < 1 h before. | None. | MI | dead |
| Baronti et al., 2022 | Italy | BNT162b2, 1^st^ dose | 76/M | 15/20 days | Unwitnessed. | None. | MI | dead |
| Baronti et al., 2022 | Italy | BNT162b2, 2^nd^ dose | 68/M | < 24 hours | Sudden death during cycling, preceded by headache and thoracic pain. | Hypertension. | MI | dead |
| Baronti et al., 2022 | Italy | mRNA1273, 1^st^ dose | 50/F | < 24 hours | Unwitnessed. | Cocaine abuse, smoking. | MI | dead |
| Fialho et al., 2022 | Portugal | ChAdOx1 vaccine (AstraZeneca, Cambridge, UK); 1^st^ dose | 59/M | 20 min | He was pale, sweaty, and had a discrete micropapular rash on his chest. | Rheumatoid arthritis, atrial fibrillation and smoking; acute coronary syndrome after influenza vaccine administration. | Type 3 KS | alive |
| Hsu et al., 2022 | Taiwan, China | ChAdOx1 nCoV- 19 vaccine; 1^st^ dose | 33/M | 9 days | 1 day of crescendo chest pain. | Obesity and mild hyperlipidemia. | STEMI | dead |
| Huang et al., 2022 | Taiwan, China | Astra-Zeneca vaccine; 1^st^ dose | 60/M | 1 hour | Chest pain and cold sweat. | Smoking and hypertension. | STEMI | alive |
| Huang et al., 2022 | Taiwan, China | ChAdOx1 nCoV-19 vaccine, AstraZeneca | 78/F | 3 days | Chest pain and dizziness. | DM2 and chronic renal failure. | STEMI | alive |
| Huang et al., 2022 | Taiwan, China | COVID-19 vaccination (AstraZeneca) | 89/M | 3 days | Progressive short of breath for 2 days. | Hypertension and DM2. | NSTEMI | alive |
| Huang et al., 2022 | Taiwan, China | COVID-19 vaccination (AstraZeneca) | 87/F | 5 days | Chest pain and progressive shortness of breath for days. | Colon cancer. | STEMI | alive |
| Iqbal et al., 2022 | PAK | Moderna COVID-19 vaccine (mRNA-1273 vaccine) ; 1^st^ dose | 61/M | 90 min | Left arm and jaw pain. | Ischemic heart disease. | NSTEMI | alive |
| Kawamura et al., 2022 | Japan | COVID-19 mRNA vaccine (Pfizer-BioNTech vaccine) ; 3^rd^ dose | 76/F | 19 hour | Chest pain. | Hypertension, dyslipidemia, and bronchial asthma. She was allergic to Japanese cedar, cypress, enicillin, and non-steroid anti-inflammatory drugs. | STEMI | alive |
| Kurozumi et al., 2022 | Japan | Pfizer-BioNTech COVID-19 vaccine; 2^nd^ dose | 70/F | 15 min | Nausea and chest pain. | Hypertension, dyslipidemia, and diabetes mellitus. | AMI | alive |
| Panthong et al., 2022 | Thailand | CoronaVac COVID-19  Vaccine; 2^nd^ dose | 48/M | 18 hour | A 30-min history of worsening nonradiated retrosternal chest pressure at rest. | He reported similar pain with less severity over the past 4 weeks since he received the first dose of the same vaccine. | NSTEMI | alive |
| Panthong et al., 2022 | Thailand | CoronaVac vaccine; 1^st^ dose | 50/M | 12 hour | An acute onset of chest discomfort at rest. | LAD artery and RCA stent lacement 4 years ago. | NSTEMI | alive |
| Şancı et al., 2022 | Turkey | COVID-19 vaccine (BNT162b2, Pfizer-BioNTech); 1^st^ dose | 22/F | 15 min | Palpitations and uneasiness of the chest. | Had previous egg and tomato allergy and no known prior drug allergies. | Type 1 KS | alive |
| This paper | China | Inactivated COVID-19 vaccine (CoronaVac, 202106061Z, Sinovac Life Sciences, Beijing, China) ; 1^st^ dose | 83/M | 10 min | Dizziness, fatigue, cold sweat and transient loss of consciousness 10-min after the 1^st^ dose of inactivated COVID-19 vaccine. Chest tightness, diarrhea and a lowest blood pressure the following day. | Subtotal gastrectomy and cholecystectomy. | AMI | alive |

**Abbreviations:** MI, myocardial infarction; AMI, acute myocardial infarction; DM2, Type 2 diabetes mellitus; NSTEMI, non-ST segment elevation myocardial infarction; STEMI, ST elevation myocardial infarction; VITT, vaccine- induced immune thrombocytopaenia and thrombosis; KS, Kounis syndrome; M, male; F, female; min: minutes.
